# Sesquiterpenes from Myrrh and Their ICAM-1 Inhibitory Activity In Vitro

**DOI:** 10.3390/molecules26010042

**Published:** 2020-12-23

**Authors:** Katrin Kuck, Guido Jürgenliemk, Bartosz Lipowicz, Jörg Heilmann

**Affiliations:** 1 Institute of Pharmaceutical Biology, University of Regensburg, Universitätsstr. 31, D-93053 Regensburg, Germany; katrin.kuck@chemie.uni-regensburg.de (K.K.); guido.juergenliemk@chemie.uni-regensburg.de (G.J.); 2 Repha GmbH Biologische Arzneimittel, Alt-Godshorn 87, D-30855 Langenhagen, Germany; bartosz.lipowicz@repha.de

**Keywords:** myrrh, *Commiphora*, ICAM-1, sesquiterpenes, seco-sesquiterpenoides

## Abstract

By using various chromatographic steps (silica flash, CPC, preparative HPLC), 16 sesquiterpenes could be isolated from an ethanolic extract of myrrh resin. Their chemical structures were elucidated by 1D and 2D NMR spectroscopy and HRESIMS. Among them, six previously unknown compounds (**1–6**) and another four metabolites previously not described for the genus *Commiphora* (**7, 10, 12, 13**) could be identified. Sesquiterpenes **1** and **2** are novel 9,10-seco-eudesmanes and exhibited an unprecedented sesquiterpene carbon skeleton, which is described here for the first time. New compound **3** is an 9,10 seco-guaian and the only peroxide isolated from myrrh so far. Compounds **1, 2, 4, 7–9, 11, 13–16** were tested in an ICAM-1 in vitro assay. Compound **7**, as well as the reference compound furanoeudesma-1,3-diene, acted as moderate inhibitors of this adhesion molecule ICAM-1 (IC_50_: 44.8 and 46.3 μM, respectively). These results give new hints on the activity of sesquiterpenes with regard to ICAM-1 inhibition and possible modes of action of myrrh in anti-inflammatory processes.

## 1. Introduction

Myrrh is a gum resin produced by plants of the genus *Commiphora* (Burseraceae), but is mainly obtained from *Commiphora myrrha* (Nees) Engl. a spinescent tree that is native to northeastern Africa, southern Arabia and India [1,2]. Besides its traditional use as incense, embalming ointment or perfume, it always had relevance as a medicinal plant and was often administered for diseases related to infection and inflammation [1,3].

In modern times, it has been shown that myrrh has antimicrobial, analgesic and anti-inflammatory activities in vitro and in vivo [1]. For instance, it is a potent inhibitor of many chemokines, cytokines and prostaglandine in vitro, which act as pro-inflammatory mediators. The essential oil of myrrh can, for example, inhibit the production of IL-1b-stimulated IL-6 and IL-8 in human gingival fibroblasts [4]. Further, ethanolic extracts were able to reduce CXCL13 and TNFα levels in activated human macrophages [5] or IL-6, IL-8, PGE_2_, MCP-1 and TNFα in a co-culture cell model of the intestinal mucosa [6], whereas an aqueous extract inhibited LPS-induced production of NO in peritoneal macrophages [7]. These effects seem to be not limited to cell culture models and have also been confirmed in vivo. It has been shown that myrrh reduces inflammatory mediators as well as the numbers of neutrophils and macrophages during cecal ligation and puncture-induced sepsis in mice and thus can increase the survival rate compared to a control group [7]. Furthermore, in an acetic acid-induced ulcerative colitis (UC) in rats, myrrh was able to decrease NO and PGE_2_ levels significantly and attenuate inflammatory processes in a concentration dependent manner [8].

In addition, the efficacy of myrrh in UC was confirmed by a double-blind, double-dummy clinical trial with a combination of myrrh, chamomile flower and coffee charcoal. This provides evidence in the maintenance therapy of remission in UC for a non-inferior application against the gold standard therapy mesalazine [9].

The pathogenesis of UC is not currently fully understood by researchers, but among other pathophysiological mechanisms adhesion molecules like the intercellular adhesion molecule 1 (ICAM-1) are supposed to play a crucial role in the inflammatory processes of inflammatory bowel diseases (IBD) [10,11,12,13]. For instance, it has been demonstrated, that the concentrations of ICAM-1 in the serum of UC patients correlates with disease activity [14].

In a healthy blood vessel ICAM-1 occurs on the apical side of endothelial cells. Triggered by cytokines like IFNγ, IL-1b, and TNFα, its expression increases during inflammation and thus enables leucocytes such as eosinophile granulocytes to migrate to the inflammation site [15]. In UC patients eosinophiles can accumulate in the gastrointestinal tract due to the increased ICAM-1 concentrations, where they can cause damage to surrounding tissue and are therefore closely involved in the development of the disease [16,17,18]. Current therapy strategies already target specific adhesion molecules. Vedolizumab, authorized for treatment of UC and Crohn’s disease, is a monoclonal antibody targeting the α4β7 integrin. Whereas Alicaforsen is an antisense oligonucleotide downregulating ICAM-1 expression, which has not been authorized yet [19].

The aim of the present study was to investigate comprehensively the phytochemical profile of myrrh extracts and to search for ICAM-1 inhibitors in an active myrrh extract, since these substances might contribute to the clinical effect the drug shows in UC related studies.

## 2. Results and Discussion

**Figure 1:**
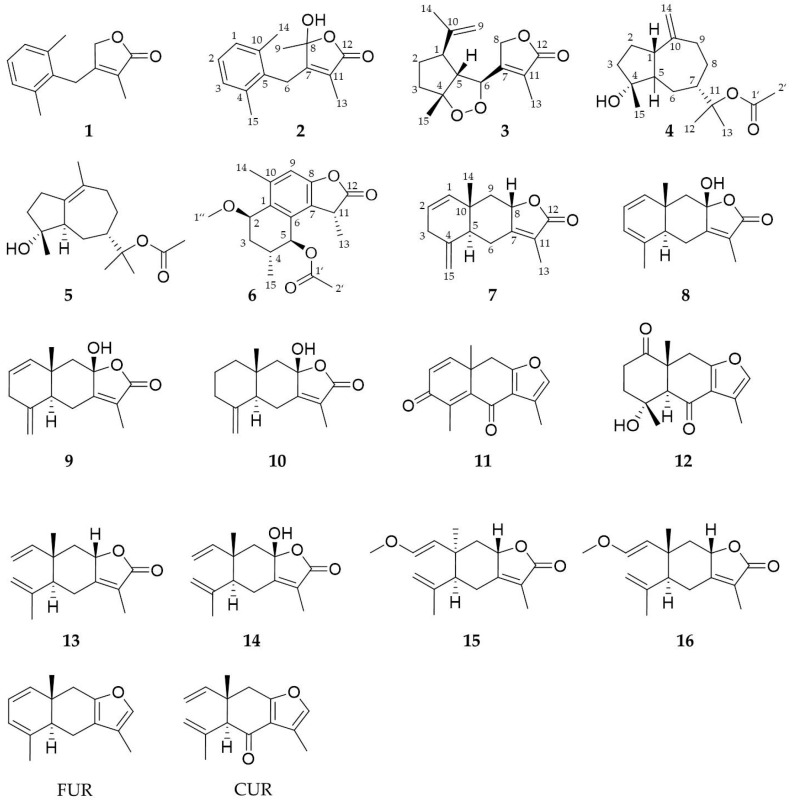
Chemical structures of the isolated sesquiterpenes (**1–16**) and the reference substances furanoeudesma-1,3-diene (FUR) and curzerenone (CUR).

By alternating maceration and percolation an ethanolic myrrh extract was prepared from the resin and portioned by liquid-liquid partition between methanol and n-heptane. Subsequently the n-heptane fraction was further separated by subsequent silica-flash, centrifugal partition chromatography (CPC) and preparative HPLC on a biphenyl column to obtain 16 sesquiterpenes (Figure 1). Compounds **1–6** are described here for the first time and four substances (**7, 10, 12, 13**) were previously unknown for the genus *Commiphora*. The isolation of compounds **9** and **14** was published before [6] as part of a characterization of a myrrh extract. Now these two substances together with recently obtained compounds and two reference substances, namely, furanoeudesma-1,3-diene (FUR) and curzerenone (CUR), are examined in an ICAM-1 in vitro assay.

### 2.1. Isolated Compounds

**Figure 2:**
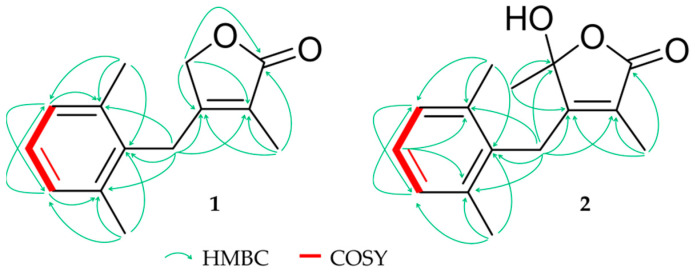
Key HMBC (heteronuclear multiple bond correlation) and COSY (correlation spectroscopy) correlations for compounds **1** and **2**.

Compound **1** (0.8 mg) was isolated as a colorless oil with an assigned formula of C_14_H_16_O_2_ on the basis of HRESIMS (*m/z* 217.1223 [M + H]^+^, calcd. for 217.1223). NMR data showed resonances of 14 carbons including three tertiary methyls (*δ_H_* 1.43 (s, H_3_-13), 2.27 (s, H_6_-14/15); *δ_C_* 11.1, 20.3, respectively), two sp^3^ methylenes (*δ_H_* 3.66 (s, H_2_-6), 4.55 (s, H_2_-8); *δ_C_* 25.7, 72.8, respectively), three aromatic methines (*δ_H_* 7.02 (d, H-1/3), 7.08 (dd, H-2); *δ_C_* 128.1, 126.7, respectively), five quaternary carbons (*δ_C_* 134.1 (C-4), 134.0 (C-10), 137.0 (C-5), 156.2 (C-7), 123.9 (C-11)) and a carbonyl carbon (*δ_C_* 175.0 (C-12)) (Table 1). Supported by 2D NMR an α,β-unsaturated γ-lactone ring as well as a second di-methylated aromatic ring was established as structural elements. The symmetry of protons and carbons in pos. 1/3, 4/10 and 14/15 together with HMBC and COSY signals (Figure 2) suggested a AB_2_ spin system in an aromatic environment namely a 2,6-dimethylphenyl moiety. This is linked to the lactone ring through C-6, which thus shares HMBC signals with both ring systems.

Compound **2** (2.1 mg) was obtained as a colorless oil and the molecular formula, which was calculated by HRESIMS as C_15_H_18_O_3_ (*m/z* 247.1333 [M + H]^+^, calcd. 247.1329) contained an additional CH_3_ and one oxygen in comparison to **1**. A first analysis of the NMR data showed similarities to compound **1** except of a missing signal for the sp^3^ methylene C-8. Instead, an oxygenated tertiary carbon (*δ_H_* 105.3 (C-8)) and a tertiary methyl (*δ_H_* 1.58 (s, H_3_-9); *δ_C_* 24.3) (Table 1) was observed, which indicated a different substitution pattern. Due to HMBC signals the proximity of the additional CH_3_ group and pos. 9 could be demonstrated adding a methyl and a hydroxyl group at the α,β-unsaturated γ-lactone ring (Figure 2).

For several sesquiterpenes the biosynthetic cleavages of C-C bonds is described resulting in seco-compounds. Thus, for example elemanes derive from eudesmanes by bond cleavage between pos. 2 and 3, but there also exist references for other seco-sesquiterpenes, which originate for example from eudesmanes [20,21,22], cadinanes [23], germacranes [23] or guaianes [24,25,26]. Thus, compounds **1** und **2** could derive from eudesmanes like **8** by cleavage of the C-C bond between pos. 9 and 10. To the best of our knowledge, this is the first report for the isolation of this unique carbon skeleton and reflecting trivial names of known sesquiterpenoides we suggest the names 9-nor-9,10-seco-isolindestrenolide (**1**) and 9,10-seco-isohydroxylindestrenolide (**2**).

**Figure 3:**
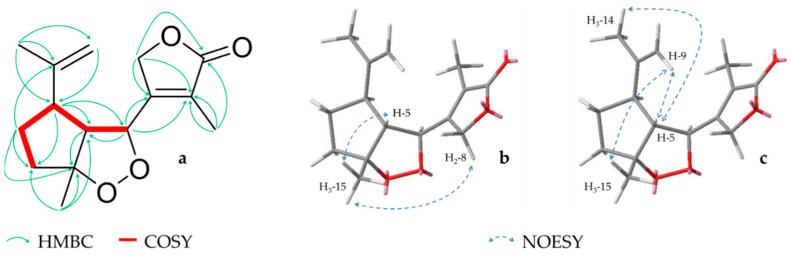
Key HMBC, COSY (**a**) and NOESY (nuclear Overhauser effect spectroscopy) correlations in the cyclic peroxide (**b**) and the cyclopentane (**c**) for compound **3**.

Compound **3** (0.7 mg) was isolated as a colorless oil. The HRESIMS suggested a molecular formula of C_15_H_20_O_4_ (*m/z* 265.1435 [M + H]^+^, calcd. 265.1434), which coincides with ^13^C spectra where 15 carbons could be detected. Further investigations of NMR data showed resonances of three tertiary methyls (*δ_H_* 1.46 (s, H_3_-15), 1.76 (s, H_3_-14), 2.22 (s, H_3_-13); *δ_C_* 24.2, 21.5, 12.8, respectively), a sp^2^ methylene (*δ_H_* 4.76/4.78 (s/brs, H-9), three quaternary carbons (*δ_C_* 123.0 (C-11), 146.0 (C-10), 161.1 (C-7)), three oxygenated positions (*δ_H_* 4.64 (s, H_2_-8), 4.79 (m, H-6); *δ_C_* 72.7, 83.4, respectively and 95.2 (C-4)) and a carbonyl carbon (*δ_C_* 172.8 (C-12)) (Table 1). Subsequent analysis of HMBC and COSY spectra revealed that **3** also possess an α,β-unsaturated γ-lactone like compound **1**, which here is linked to a cyclopentane ring. Due to HMBC signals to both ring systems, pos. 6 seemed to form the conjunction between these two structure elements. Furthermore, a methyl and an isopropenyl group could be established as substituents of the cyclopentyl ring and the position of the oxygenated carbons was ascertained in pos. 4 and 6. Nevertheless, the formula determined by HRESIMS showed one more degree of unsaturation and due to the fact that all other atoms could be unambiguously assigned, a closure of the cyclic peroxide from pos. 4 to 6 could be implied (Figure 3a). Additionally, the relative configuration was determined by investigation of NOESY spectra, which showed signals between the methyl group (H_3_-15) and the protons H-5 and H_2_-8. Thus, it could be assumed that these elements are located at one side of the cyclic oxide (Figure 3b). Furthermore, correlations between pos. 5, 15 and the isopropyl protons (H-9, -14) could also be observed and indicated an identical orientation in pos. 1 (Figure 3c), which implied a *rel-1R,4R,5R,6S* configuration.

Endoperoxid substructures have been rarely found among secondary plant metabolites, but are also present in other sesquiterpenes like artemisinine. Furthermore, the biosynthetic origin of the carbon skeleton of compound **3** can be explained by a C-C cleavage between pos. 8 and 9 in a guaiane type sesquiterpene. In the literature, other seco-guaianolides were previously described mostly in the genus *Tanacetum* and *Artemisia*. The majority of these compounds belong to the 1,10-seco-type [24,25,27,28,29], but also 4,5-seco-molecules do occur [26,30] whereas a 8,9 C-C cleavage has only once been reported for *Curcuma wenyujin* [31]. Thus, describing a peroxide in myrrh for the first time, we suggest the name myrrhanoperoxide.

**Figure 4:**
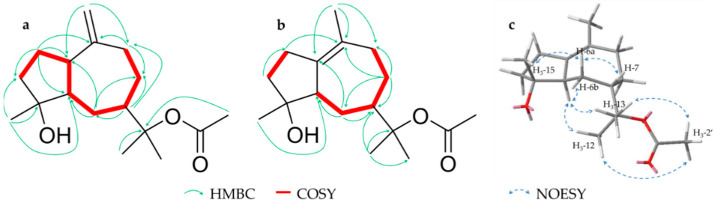
Key HMBC and COSY correlations for compound **4** (**a**) and **5** (**b**) as well as relevant NOESY signals of compound **5** (**c**).

Compound **4** (6.9 mg), a colorless oil, was assigned to a molecular formula of C_17_H_28_O_3_ on the basis of HRESIMS (*m/z* 303.1931 [M + Na]^+^, calcd. for 303.1931). NMR data showed 17 carbons including an acetyl substituent (*δ_H_* 1.97 (s, H_3_-2′); *δ_C_* 22.6 (C-2′) and 170.5 (C-1′)) as well as three tertiary methyls (*δ_H_* 1.41 (s, H_3_-12/13), 1.20 (s, H_3_-15); *δ_C_* 23.2, 23.4, 23.8, respectively), an oxygenated quaternary carbon (*δ_C_* 81.2 (C-4)) and two sp^2^ hybridized positions (*δ_H_* 4.68/4.70 (s/s, H-14); *δ_C_* 106.3 (C-14) and 153.1 (C-10)) indicating a terminal double bond (Table 2). Secondary analyses of 2D data suggested that **4** is a guaiane type sesquiterpene with an olefinic double bond between C-10 and C-14, a hydroxylation at C-4 and an acetylation at C-11. Key HMBC and COSY correlations are shown in Figure 4a. Due to NOESY signals between H-1, H-15, H-6a (*δ_H_* 1.51) and H-7 these protons could be located on one side of the ring level, whereas H-6b (*δ_H_* 1.64) and the acetylated isopropyl structure (H-12/13) form a signal on the other side. Thus, the relative configuration *1S,4R,7S* was established while the relative stereochemistry at pos. 5 could not be determined due to interference of H-5 with other protons. Literature research showed that a structure with the same constitution has been previously isolated [32] whereby a *1R,4R,5R* configuration was postulated for a [α]_D_ = +20 Although the few published NMR shifts match those of **4**, it is excluded that the two compounds are identical because of distinct NOESY signals and a deviating ${[\alpha ]\;}_{D}^{25}$ (+38.2). Therefore, reflecting trivial names of known guaianes, we suggest the name *rel*-(+)-(*1S,4R,7S)*-11-acetyl-guai-10(14)-en-4,11-ol for **4**.

Compound **5** (4.1 mg) is an isomer of **4** with the same molecular formula C_17_H_28_O_3_ (HRESIMS, *m/z* 303.1930 [M + Na]^+^, calcd. for 303.1931) and was also obtained as a colorless oil. A review of NMR data showed similar characteristics and shifts except for the sp^2^ methylene (C-14), which was missing and replaced by an additional tertiary methyl (*δ_H_* 1.55 (s, H_3_-14); *δ_C_* 20.9) (Table 2). This indicated a shift of the double bond from pos. 10/14 to 1/10, which could be confirmed by HMBC and COSY correlations (Figure 4b). Additionally, the position of the acetyl group could be established by a NOESY correlation between H_3_-2′ and the two methyl groups in pos. 12/13. Furthermore, in contrast to **4**, the complete relative configuration could be determined by NOESY signals above the ring level from H-6a (*δ_H_* 1.51) to H-7 and -15 and below from H-5 to H-6b (*δ_H_* 1.72) and the two methyl groups H-12/13 (Figure 4**c**). Thus, a relative stereochemistry of *4R,5R,7S* can be implied and we suggest the name *rel*-(+)-(*4R,5R,7S)*-11-acetyl-guai-1(10)-en-4,11-ol in analogy to **4**.

**Figure 5:**
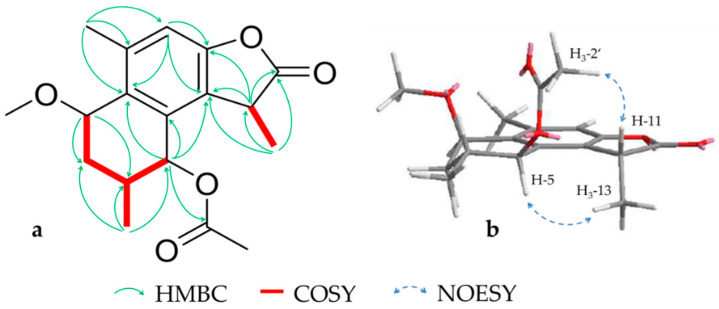
Key HMBC, COSY (**a**) and NOESY (**b**) correlations for compound **6**.

Compound **6** (0.9 mg) was isolated as a colorless oil and assigned a molecular formula of C_18_H_22_O_5_ by HRESIMS (*m/z* 341.1359 [M + Na]^+^, calcd. 341.1359). According to NMR data, the structure contained an aromatic system consisting of five quaternary carbons (*δ_C_* 124.0 (C-7), 131.7 (C-1), 133.0 (C-6), 140.3 (C-10), 153.4 (C-8)) and a sp^2^ methine (*δ_H_* 6.96 (s, H-9); *δ_C_* 112.9) as well as a methoxy (*δ_H_* 3.45 (s, H_3_-1′); *δ_C_* 56.4) and an acetyl substituent (*δ_H_* 2.17 (s, H_3_-2′); *δ_C_* 20.9 (C-2′) and 171.1 (C-1′)). Additionally, three tertiary methyls ((*δ_H_* 1.09 (d, H_3_-15), 1.47 (d, H_3_-13), 2.39 (s, H_3_-14); *δ_C_* 19.1, 15.5, 19.5, respectively), two oxygenated carbons (*δ_H_* 4.30 (dd, H-2), 5.87 (d, H-5); *δ_C_* 73.1, 74.9, respectively) and a carbonyl carbon (*δ_C_* 178.3 (C-12)) were found (Table 3). The structure showed similarities to previously isolated commiterpene B from *C. myrrha* [33] including the substitution pattern and relative configuration, but the signals of H-11 and H-13 were split to a quartet and a doublet and thus shared a coupling constant of 7.4 Hz. This indicated that here the furan ring of commiterpene B is oxygenated to a γ-lactone as in case of other cadinane type sesquiterpenlactones from *Chloranthus henryi* [34] (Figure 5a). To determine the orientation of the so emerged additional stereo center in pos. 11, the NOESY signals of the substituents were compared with those of pos. 5. An *R* configuration could be implied due to the strong correlation between the H-11 and the acetyl group as well as H-5 to H-13 (Figure 5b). Referring to the nomenclature started by Xu et al. [33], we suggest the name commiterpene D for compound **6**.

**Figure 6:**
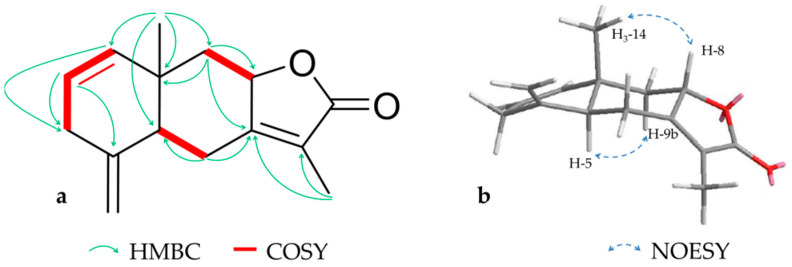
Key HMBC, COSY (**a**) and NOESY (**b**) correlations for compound **7**.

Compound **7** (6.6 mg) was obtained as a colorless oil and assigned a molecular formula of C_15_H_18_O_2_ by HRESIMS (*m/z* 231.1382 [M + H]^+^, calcd. for 231.1380). According to NMR data **7**, was identified as previously isolated lindestrenolide [35], which was described 1964 by Takeda et al. as a constituent of *Lindera strychnifolia* Vill. Due to the fact that the characterization of this compound in the literature is insufficient, a complete set of NMR data is here reported for the first time (shown in Figure 6 and Table 3). Key to structure elucidation were HMBC correlations and three independent spin systems, which indicated a typical eudesmanolide. Additional NOESY signals between H-9a (*δ_H_* 2.37) and -5 as well as between H-8 and H-14 revealed a *5S,8S,10S* configuration as described in the literature.

**Figure 7:**
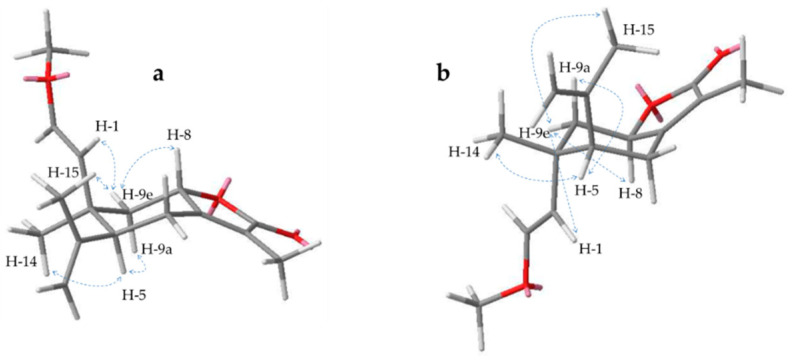
Key NOESY correlations for compound **15** in *rel-5S,8S,10S* (**a**) and *rel-5S,8R,10R* (**b**) configuration.

In the literature the diastereomeres **15** and **16** were isolated in a 1:1 mixture and characterized as 2-methoxyisogermafurenolide and 8-epi-2-methoxyisogermafurenolide with a configuration of *rel-5S,8R,10R* and *rel-5S,8S,10R*, respectively [36]. Now these compounds could be separated for the first time by preparative HPLC on a biphenyl column, which allowed a more precise assessment of their stereochemistry by NOESY spectra. Thus, the configuration of **16** could be confirmed, while the new data indicated a different relative stereochemistry for **15.** This was suggested by correlations under the ring level from H-5 to -9a and the methyl group at pos. 14 as well as above between H-9e and -1, -8 and -15 (Figure 7). In addition, signals from one side of the ring to the other were missing or much weaker. Therefore, a *rel-5S,8S,10S* configuration for **15** seems much more likely, defining this compound as 10-epi isomer of **16**, namely, 10-epi-2-methoxyisogermafurenolide.

Other compounds were identified according to literature as lindestrenolide (**7**) [35], isohydroxylindestrenolide (**8**) [36], hydroxylindestrenolide (**9**) [35,37], atractylenolide III (**10**) [38], commiphorane E3 (**11**) [39], 4β-hydroxy-8,12-epoxyeudesma-7,11-diene-1,6-dione (**12**) [40], isogermafurenolide (**13**) [35], hydroxyisogermafurenolide (**14**) [35,37] and 2-methoxyiso- germafurenolide (**15**) [36].

### 2.2. Purity and ICAM-1 Inhibition

**Figure 8:**
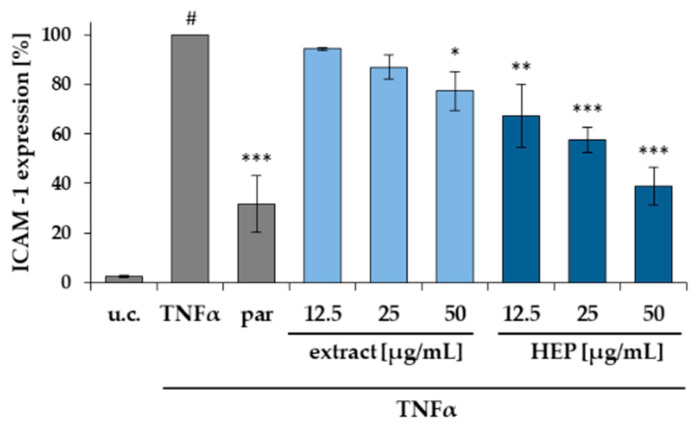
Influence of the ethanolic extract and the n-heptane fraction (HEP) on ICAM-1 expression in HMEC-1 cells. The test was performed with pure medium (u.c.), with TNFα (10 ng/mL) and parthenolide + TNFα (par, 5 μM) as positive control. Substance concentrations between 12.5–50 μg/mL were applied. Data are presented as mean ± SD; # *p* < 0.001 vs. u.c.; * *p* < 0.05, ** *p* < 0.01, *** *p* < 0.001 vs. TNFα (*n* = 3).

To examine the activity of myrrh, the ethanolic extract and an HEP fraction (n-heptane fraction) were tested in an in vitro assay to monitor the TNFα dependent expression of ICAM-1 in HMEC-1 cells. The assay has been performed as previously described [41]. In brief, the assay was carried out using an untreated control (u.c.), a negative control with TNFα (10 ng/mL), referred to as 100% value and a positive control with parthenolide (5 μM). Whereas the extract showed a moderate effect, the HEP fraction was able to cause a significant inhibition of ICAM-1 expression in a concentration depended manner (Figure 8). All tested concentrations were also investigated in a MTT assay for their cytotoxic effect on HMEC-1 cells and the mean viability was within a range of 95–105% (Figure S1).

**Figure 9:**
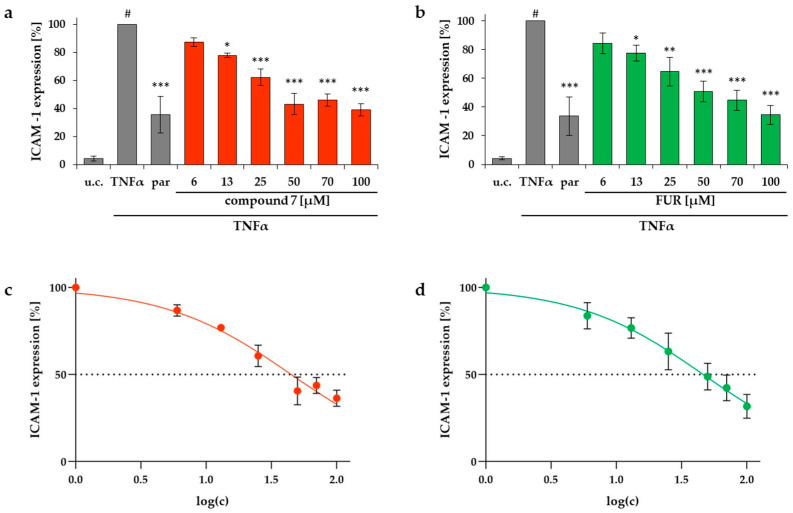
Influence of compound **7** (**a**,**c**) and FUR (**b**,**d**) on ICAM-1 expression in HMEC-1 cells. The test was performed with pure medium (u.c.), with TNFα (10 ng/mL) and parthenolide + TNFα (par, 5 μM) as positive control. Substance concentrations between 6–100 μM were applied. Data are presented as mean ± SD (**a**,**b**) and nonlinear regression curve (**c**,**d**); # *p* < 0.001 vs. u.c.; * *p* < 0.05, **p < 0.01, *** *p* < 0.001 vs. TNFα (*n* = 3).

Compounds that could be obtained in sufficient purity (>90%) and amount, **1, 2, 4, 7–9, 11, 13–16** as well as two reference substances (FUR and CUR, Figure 1) from *C. myrrha* were also tested in the same assay. Despite of the structural resemblance of all the tested substances, only two of them were able to reduce the ICAM-1 expression in a significant manner. Compound **7** as well as furanoeudesma-1,3-diene showed a concentration dependent effect in this test with IC_50_ values of 44.8 and 46.3 μM, respectively (Figure 9). All tested concentrations were investigated in an MTT assay for their effect on HMEC-1 cells and the mean viability was within a range of 95–105% (Figure S1).

Several sesquiterpenlactones like helenalin [42] and parthenolide [43] are known for their ability to inhibit the central transcription factor NF-κB, which is linked to TNFα. The mechanism of this interaction has been intensely investigated and affiliated to α,β-unsaturated carbonyl structures, for example α-methylene-γ-lactones or α,β-unsubstituted cyclopentenones which can cause a Michael like addition of sesquiterpene lactones to SH-groups of NF-κB [44,45,46]. This alkylation prevents the transcription factor of binding to the DNA and enhance the production of proinflammatory mediators and effectors like ICAM-1 [47].

All compounds tested didn’t contain α-methylene-γ-lactones and from the α,β-unsubstituted cyclopentenone substructures in **11** and **12** a potent Michael activity cannot be expected [48]. Thus, activity of **7** and FUR is somewhat surprising. Remarkably, all tested substances exhibiting a hydroxyl group in pos. 8 such as **8, 9** and **14** did not show any activity in the assay although other structural elements remained unaltered. A similar observation was made in a quantitative structure-activity relationship study, which showed that the number of hydroxyl groups in a sesquiterpene lactone has a negative effect on its inhibitory effect on NF-κB [49]. Weather this is the result of an interference with the mode of action or the hydroxyl groups just prevents the molecule of reaching the cytosol through lipophilic membranes remains unclear. Furthermore, cleavages between pos. 9 and 10 (seco-eudesmanes **1** and **2**) or 2 and 3 (elemanes **13–16**) seem to result in a loss of activity even though the lipophilicity remains alike.

In contrast to compound **7**, furanoeudesma-1,3-diene is missing the lactone ring, which is replaced by a 3-methylfuran. Therefore, an alkylation of NF-κB as mode of action of furanoeudesma-1,3-diene seems also unlikely. Curzerenone also did not show any activity due to the fact, that the carbonyl structure is located next to the furan ring and therefore not accessible for a Michael like addition.

To our knowledge, this is the first report showing an activity of myrrh compounds towards an ICAM-1 inhibition. ICAM-1 expression is known to be upregulated in IBD and considered as a possible target for UC [19]. UC aetiology is not fully understood but the current understanding is that different factors lead to an inadequate immune response and intestinal barrier impairment [11,12,13]. Myrrh extracts have already shown activity in in vitro and in vivo tests aiming towards this inadequate immune response and intestinal barrier impairment [5,6,8,50]. This study hints towards a new possible mechanism for myrrh or myrrh constituents in IBD emphasizing a multimodal activity of myrrh.

## 3. Materials and Methods

### 3.1. Chemicals

Ethanol for extraction was purchased in technical quality from CSC Jäcklechemie (Nürnberg, Germany) and purified by evaporation. Methanol, n-heptane, ethyl acetate, dichloromethane diethyl ether and toluene (all p.a.) were obtained from Fisher Scientific (Hampton, NH, USA) whereas sea sand (technical), anisaldehyde (4-methoxybenzaldehyd for synthesis), sulfuric acid (95–97%, p.a.) and acetonitrile (HPLC-grade) were provided by Merck Chemicals (Darmstadt, Germany) and *n*-hexane (p.a)., acetic acid (p.a.), chloroform-d (99.8%), TNFα (≥97%, recombinant, human, *E. coli*) and Dulbecco’s Phosphate Buffered Saline by Sigma-Aldrich (St. Louis, MO, USA). Methanol-d_4_ (methylalcohol-d_4_) was bought from Deutero (Kastellaun, Germany) and formic acid (p.a.) and DMSO (p.a. ≥ 99.5% for molecular biology) from Carl Roth (Karlsruhe, Germany). Other cell culture supplies like Easy Endothelial Cell Growth Medium and supplement-mix were purchased at PeloBiotech (Planegg, Germany), Biochrom (Berlin, Germany) (FBS Superior, trypsin/EDTA), Bio-Rad (Feldkirchen, Germany) (murine FITC-marked monoclonal antibody (MCA1615F)) or Calbiochem (Bad Soden, Germany) (parthenolide, <97%). Formalin (10%, phosphate buffered) was obtained by AppliChem (Darmstadt, Germany).

### 3.2. Plant Material and Extraction

Powdered myrrh resin of *C. myrrha* (Myrrha, Ph. Eur. 2016) was provided by Lomapharm^®^ (lot NM0160, Rudolf Lohmann GmbH KG, Emmerthal, Germany). 3 kg powdered resin was mixed with 4.5 kg sea sand and extracted over seven days by alternating percolation and maceration with 26 L ethanol 96% (*v*/*v*). The extract was dried by evaporation and lyophilisation yielding a total amount of 761.65 g (DER: 3.9:1) and was stored light protected at −20 °C.

### 3.3. Isolation

#### 3.3.1. Liquid–liquid Partition

Four portions (100 g) of ethanolic extract were solved in 1 L methanol each and partitioned eight times with 0.5 L n-heptane in a separatory funnel. Subsequently the combined methanol (MeOH) and *n*-heptane (HEP) portions were dried by evaporation and lyophilisation and were stored light protected at −20 °C. The total amounts gained during this process add up to 328.13 g (MeOH) and 69.99 g (HEP).

#### 3.3.2. Silica Flash 1

A Spot flash system (Armen Instrument, Paris, France) equipped with a SVP D40 silica column (13 × 4 cm, SI60 15–40 μm, 90 g, Götec Labortechnik GmbH, Bickenbach, Germany) was used. 2 g HEP fraction were solved in starting conditions (50% *m*/*v*) and injected on the equilibrated column. The gradient between *n*-hexane (A) and ethylacetate (B) was carried out with a flow of 30 mL/min as follows: 0–30 min 5% B, 30-90 min 5–20% B, 90–110 min 20–100% B, 110–140 min 100% B. Thereby, 10 fractions (F1–10) were collected according to TLC control of which F5–7 (1301–1875 mL; 1876–2450 mL; 2451–2950 mL) were further investigated. The process was repeated ten times and the fractions pooled and dried by evaporation to gather 2.4905 g F5, 1.1153 g F6 and 1.6045 F7.

#### 3.3.3. CPC (Centrifugal Partition Chromatography)

CPC was performed on a Spot CPC device with a 250 mL rotor (Armen Instrument, Paris, France), a 510 HPLC pump (Waters GmbH, Eschborn, Germany) and a 2111 Multirac Fraction Collector (LKB-Produkter AB, Bromma, Sweden). Prior to separation, a solvent system consisting of n-hexane, acetonitrile and methanol (40/25/10 *v*/*v*/*v*) [51] was equilibrated in a separatory funnel and the two phases were separated before analysis and degassed for 10 min. Subsequently the rotor was filled with lower phase (LP) and then loaded with upper phase (UP) in ascending mode (ASC) with a rotation speed of 1000 rpm and a flow of 5 mL/min. 1.0–1.5 g of F5–7 were solved in a mixture of UP/LP (50/50, *v*/*v*), injected in the equilibrated system and fractions of 5 mL were collected. After 800 mL the mode was switched to descending mode (DSC) and the system purged with 200 mL (LP). For F5 the process war repeated once. Following, subfractions (F5C1-6, F6C1-8 and F7C1-8) were formed according to TLC control and dried by evaporation for further use. F5: F5C5 (DSC 21–135 mL; 641.3 mg), F6: F6C7 (DSC 16–190 mL; 386.2 mg), F7: F7C6 (DSC 51–110 mL; 124.2 mg), F7C7 (DSC 11–50 mL; 508.4 mg).

#### 3.3.4. Silica Flash 2

F6C7 and F7C7 were submitted to a second Flash separation using the same system and a SVF D26 silica column (9 × 2.8 cm, SI60 15–40 μm, 30 g, Götec Labortechnik GmbH, Bickenbach, Germany). Analogously to Section 3.3.2, 250–350 mg sample were processed with 15 mL/min, a fraction size of 15 mL and the following gradient consisting of dichloromethane (A) and ethyl acetate (B): 0–60 min 1–6% B, 60–90 min 6–15% B, 90–92 min 15–100% B, 92–110 min 100% B. For F7C7 the process was repeated once. Following, subfractions (F6C7F1-5 and F7C7F1-5) were formed according to TLC control and dried by evaporation for further use. F6C7F1 (0–165 mL; 24.0 mg), F6C7F2 (166–255 mL; 64.5 mg), F6C7F4 (511–1410 mL; 142.5 mg), F7C7F3 (466–660 mL; 52.6 mg), F7C7F4 (661-1410 mL, 174.0 mg).

#### 3.3.5. TLC (Thin Layer Chromatography)

Fraction control by TLC was carried out for Flash chromatography and CPC on silica gel 60 F254 (Merck, Darmstadt, Germany) with a mobile phase consisting of toluene and ethylacetate (95/5, *v*/*v*). Plates were derivatized with anisaldehyde reagent R and a Camag TLC visualizer was used for documentation (Camag AG, Muttenz, Switzerland).

#### 3.3.6. Preparative HPLC (High-Performance Liquid Chromatography)

An preparative HPLC equipped with a 1260 Infinity binary pump, a 1260 Infinity manual injector, a 1260 Infinity fraction collector, a 1260 Infinity diode array detector (all Agilent Technologies, Santa Clara, CA, USA) and a Kinetex^®^ column (Biphenyl, 100 Å, 5 μm, 21.2 × 250 mm, Phenomenex, Aschaffenburg, Germany) was used. Samples were solved in acetonitrile and portions of 0.2–5 mg were injected manually following a separation with acetonitrile (A) / water (B) and a flow of 21 mL/min. Thereby, peaks were detected at 200 nm, collected manually and pooled. After elimination of acetonitrile via evaporation the water fractions were partitioned four times with diethyl ether and the organic phases were dried in a nitrogen flow. For separation, the following gradients were used to collect the mentioned isolates: F5C5 (0–16 min 45–53% B, 16–17 min 53–100% B, 17–22 min 100% B; RT 18.2 min (**7**) and 19.4 min (**13**)); F6C7F1 (0–19 min 35–55% B, 19–25 min 55–100% B, 25–30 min 100% B; RT 13.4 min (**1**), RT 14.2 min (**3**)); F6C7F2 (0–23 min 30–54% B, 23–24 min 54–100% B, 24–30 min 100% B; RT 15.8 min (**11**), RT 21.4 min (**15**); RT 22.2 min (**16**), RT 22,9 min (**4**)); F6C7F4 (0–12 min 38–62% B, 12–20 min 62–100% B, 20–21 min 100% B; RT 6.2 min (**12**)); F7C6 (0–26 min 30–45% B, 26–27 min 45–100% B, 27–32 min 100% B; RT 23.2 min (**5**), RT 25.4 min (**6**)); F7C7F3 (0–19 min 25–45% B, 19–20 min 45–100% B, 20–25 min 100% B; RT 20.1 min (**2**)); F7C7F4 (0–17 min 30–48% B, 17–18 min 48–100% B, 18–23 min 100% B; RT 15.6 min (**8**), RT 16.0 min (**9**), RT 17.0 min (**14**), RT 17.4 min (**10**)).

### 3.4. NMR

Samples were solved in CDCl_3_ or methanol-d_4_, transferred to 507-HP-8 NMR tubes (Norell Inc, Morganton, NC, USA) and analyzed with an AVANCE III 600 NMR equipped with a 5 mm TBI CryoProbe (^1^H-NMR 600.25 MHz, ^13^C-NMR 150.95 MHz, 298 K) or an AVANCE III HD NMR (^1^H-NMR 400.13 MHz, ^13^C-NMR 100.63 MHz, 299 K) (Bruker Corporation, Billerica, MA, USA). Subsequently structures were elucidated on basis of 1D-^1^H, 1D-^13^C as well as 2D-^1^H,^13^C HSQC, ^1^H,^13^C HMBC, ^1^H,^1^H COSY and ^1^H,^1^H NOESY experiments with TopSpin 3.5.b.91 pl 7 (Bruker Corporation).

### 3.5. UHPLC-MS

Isolates were analyzed by UHPLC-MS (1290 Infinity UHPLC and Q-TOF 6540 UHD, Agilent Technologies, Santa Clara, CA, USA) on a ZORBAX Eclipse column (XDB-C18 RRHD, 2.1 × 100 mm, 1.8 µm, Agilent Technologies) with solvent A (water with 0.1% formic acid) and solvent B (acetonitrile with 0.1% formic acid). gradient: 0–10 min, 20–98% B; 10–12 min, 98% B; 12–12.1 min, 98–20% B; 12.1–13.5 min, 20% B with a flow of 0.5 mL/min, a column temperature of 50 °C and an injection volume of 1 µl. Subsequently MS analysis was performed with electrospray ionization (ESI) in positive and negative mode.

### 3.6. Optical Methods

All optical data were determined using solutions in methanol. Specific optical rotations were recorded at an UniPol L 1000 polarimeter (Schmidt + Haensch GmbH & Co., Berlin, Germany) using a micro tube (50 mm, 550 μL) at 589 nm. UV-spectra were measured on a Cary 50 Scan UV-spectrophotometer (Varian Deutschland GmbH, Darmstadt, Germany) in a quartz cuvette (QS, 1.0 cm, Hellma GmbH and Co. KG, Müllheim, Germany) in a range of 200–800 nm. For CD spectra a J-715 spectropolarimeter (JASCODeutschland GmbH, Gross-Umstadt, Germany) was used with a 0.1 cm quartz cuvette (Type: 100-QSQ, Hellma GmbH and Co. KG). Each measurement was repeated ten times at 22 °C from 190–300 nm with a scanning rate of 200 nm/min in 0.5 nm steps. Savitzky–Golay algorithm was used for spectra smoothing (convolution width: 15).

### 3.7. Purity

The purity of isolates was determined by HPLC-DAD (190–400 nm) analysis using an Elite LaChrom system consisting of an autosampler L-2200, a pump L-2130, an column oven L-2350, a diode array detector L-2455 (all Hitachi, Tokyo, Japan) and a Kinetex^®^ biphenyl column (100 Å, 5 µm, 4.6 × 250 mm, Phenomenex, Aschaffenburg, Germany). The gradients described in Section 3.3.6 were used to analyze 5 µL (acetonitrile, 1 mg/mL) with a flow of 1 mL/min and the chromatograms processed with EZChrom Elite 3.1.7 (Hitachi). Thus, the purity was calculated as the proportion of the integral of the main peak in the chromatogram using the maxplot (adjusted by a blank).

### 3.8. Isolates

#### 3.8.1. 9-Nor-9,10-seco-isolindestrenolide (**1**)

A total of 0.8 mg, colorless oil; UV (MeOH) λ_max_ (log ε): 202.0 (3.97), 280.9 (2.41); ^1^H and ^13^C-NMR data (CDCl_3_, 600 and 150 MHz, respectively) in Table 1; HRESIMS *m/z* 217.1223 [M + H]^+^ (calcd. for C_14_H_16_O_2_, 217.1223); test purity according to Section 3.7. 98.9%.

#### 3.8.2. 9,10-Seco-isohydroxylindestrenolide (**2**)

A total of 2.1 mg, colorless oil; ${[\alpha ]\;}_{D}^{25}$ -2 (*c* 0.22, MeOH); UV (MeOH) λ_max_ (log ε): 210 (4.10), 282.0 (2.93); CD: Figure S2; ^1^H and ^13^C-NMR data (CDCl_3_, 600 and 150 MHz, respectively) in Table 1; HRESIMS *m/z* 247.1333 [M + H]^+^ (calcd. for C_15_H_18_O_3_, 247.1329); test purity according to Section 3.7. 95.6%.

#### 3.8.3. Myrrhanoperoxide (**3**)

A total of 0.7 mg, colorless oil; ${[\alpha ]\;}_{D}^{25}$ +17 (*c* 0.07, MeOH); UV (MeOH) λ_max_ (log ε): 205.9 (3.86); CD: Figure S2; ^1^H and ^13^C-NMR data (CDCl_3_, 600 and 150 MHz, respectively) in Table 1; HRESIMS *m/z* 265.1435 [M + H]^+^ (calcd. for C_15_H_20_O_4_, 265.1434).

#### 3.8.4. *rel*-(+)-(*1S,4R,7S*)-11-Acetyl-guai-10(14)-en-4,11-ol (**4**)

A total of 6.9 mg, colorless oil; ${[\alpha ]\;}_{D}^{25}$ +38 (*c* 0.24, MeOH); UV (MeOH) λ_max_ (log ε): 202.0 (3.68); CD: Figure S2; ^1^H and ^13^C-NMR data (CDCl_3_, 400 and 100 MHz, respectively) in Table 2; HRESIMS *m/z* 303.1931 [M + Na]^+^ (calcd. for C_17_H_28_O_3_, 303.1931); test purity according to Section 3.7. 93.7%.

#### 3.8.5. *rel-*(+)-(*4R,5R,7S*)-11-Acetyl-guai-1(10)-en-4,11-ol (**5**)

A total of 4.1 mg, colorless oil; ${[\alpha ]\;}_{D}^{25}$ +32 (*c* 0.22, MeOH); UV (MeOH) λ_max_ (log ε): 203.0 (3.73); CD: Figure S2; ^1^H and ^13^C-NMR data (CDCl_3_, 400 and 100 MHz, respectively) in Table 2; HRESIMS *m/z* 303.1930 [M + Na]^+^ (calcd. for C_17_H_28_O_3_, 303.1931).

#### 3.8.6. Commiterpene D (**6**)

A total of 0.9 mg, colorless oil; ${[\alpha ]\;}_{D}^{25}$ -8 (*c* 0.12, MeOH); UV (MeOH) λ_max_ (log ε): 204.1 (4.11), 276.1 (3.29); CD: Figure S2; ^1^H and ^13^C-NMR data (CDCl_3_, 600 and 150 MHz, respectively) in Table 3; HRESIMS *m/z* 341.1359 [M + H]^+^ (calcd. for C_18_H_22_O_5_, 341.1359).

#### 3.8.7. Lindestrenolide (**7**)

A total of 6.6 mg, colorless oil; ${[\alpha ]\;}_{D}^{25}$ +96 (*c* 0.23, MeOH); UV (MeOH) λ_max_ (log ε): 219.0 (4.04), 263.9 (3.07); CD: Figure S2; ^1^H and ^13^C-NMR data (MeOD, 400 and 100 MHz, respectively) in Table 3; HRESIMS *m/z* 231.1382 [M + H]^+^ (calcd. for C_15_H_18_O_2_, 231.1380); test purity according to Section 3.7. 97.5%.

#### 3.8.8. Isohydroxylindestrenolide (**8**)

A total of 7.4 mg, white crystals; ${[\alpha ]\;}_{D}^{25}$ +57 (*c* 0.18, MeOH)¸ UV (MeOH) λ_max_ (log ε): 215.1 (4.14), 263.9 (3.68); ^1^H-NMR data (CDCl_3_, 400 MHz) in Table S1,^13^C-NMR data (CDCl_3_, 100 MHz) in Table S3; HRESIMS *m/z* 247.1330 [M + H]^+^ (calcd. for C_15_H_18_O_3_, 247.1329); test purity according to Section 3.7. 94.9%.

#### 3.8.9. Hydroxylindestrenolide (**9**)

A total of 13.3 mg, white crystals; ${[\alpha ]\;}_{D}^{25}$ +195 (*c* 0.21, MeOH); UV (MeOH) λ_max_ (log ε): 218.1 (4.11); CD: Figure S2; HRESIMS *m/z* 247.1334 [M + H]^+^ (calcd. for C_15_H_18_O_3_, 247.1329); test purity according to Section 3.7. 98.7%.

#### 3.8.10. Atractylenolide (**10**)

A total of 2.8 mg, white crystals; ${[\alpha ]\;}_{D}^{25}\;$+7 (*c* 0.17, MeOH); UV (MeOH) λ_max_ (log ε): 219.0 (3.82); CD: Figure S2; ^1^H-NMR data (CDCl_3_, 600 MHz) in Table S1, ^13^C-NMR data (CDCl_3_, 150 MHz) in Table S3; HRESIMS *m/z* 249.1490 [M + H]^+^ (calcd. for C_15_H_20_O_3_, 249.1485).

#### 3.8.11. Commiphorane E3 (**11**)

A total of 1.6 mg, yellow crystals; ${[\alpha ]\;}_{D}^{25}$ -33 (*c* 0.20, MeOH); UV (MeOH) λ_max_ (log ε): 246.9 (4.08), 308.7 (3.51); CD: Figure S2; ^1^H-NMR data (CDCl_3_, 600 MHz) in Table S1, ^13^C-NMR data (CDCl_3_, 150 MHz) in Table S3; HRESIMS *m/z* 243.1015 [M + H]^+^ (calcd. for C_15_H_14_O_3_, 243.1016); test purity according to Section 3.7. 94.8%.

#### 3.8.12. 4β-Hydroxy-8,12-epoxyeudesma-7,11-diene-1,6-dione (**12**)

A total of 1.7 mg, white crystals; ${[\alpha ]\;}_{D}^{25}$ +14 (*c* 0.17, MeOH); UV (MeOH) λ_max_ (log ε): 203.0 (4.11), 267,9 (3.31); CD: Figure S2; ^1^H-NMR data (CDCl_3_, 600 MHz) in Table S1, ^13^C-NMR data (CDCl_3_, 150 MHz) in Table S3; HRESIMS *m/z* 263.1279 [M + H]^+^ (calcd. for C_15_H_18_O_4_, 263.1278).

#### 3.8.13. Isogermafurenolide (**13**)

A total of 0.8 mg, white crystals; ${[\alpha ]\;}_{D}^{25}$ +14 (*c* 0.10, MeOH); UV (MeOH) λ_max_ (log ε): 219.9 (3.76); CD: Figure S2; ^1^H-NMR data (CDCl_3_, 600 MHz) in Table S2, ^13^C-NMR data (CDCl_3_, 150 MHz) in Table S3; HRESIMS *m/z* 233.1539 [M + H]^+^ (calcd. for C_15_H_20_O_2_, 233.1536); test purity according to Section 3.7. 91.0%.

#### 3.8.14. Hydroxyisogermafurenolide (**14**)

A total of 6.3 mg, white crystals; ${[\alpha ]\;}_{D}^{25}$ +3 (*c* 0.21, MeOH); UV (MeOH) λ_max_ (log ε): 218.1 (3.95); CD: Figure S2; HRESIMS *m/z* 249.1487 [M + H]^+^ (calcd. for C_15_H_20_O_3_, 249.1485); test purity according to Section 3.7. 97.9%.

#### 3.8.15. 8-Epi-2-methoxyisogermafurenolide (**15**)

A total of 1.2 mg, white crystals; ${[\alpha ]\;}_{D}^{25}$ +54 (*c* 0.15, MeOH); UV (MeOH) λ_max_ (log ε): 217.0 (4.07); CD: Figure S2; ^1^H-NMR data (CDCl_3_, 600 MHz) in Table S2, ^13^C-NMR data (CDCl_3_, 150 MHz) in Table S3; HRESIMS *m/z* 263.1642 [M + H]^+^ (calcd. for C_16_H_22_O_3_, 263.1642); test purity according to Section 3.7. 95.6%

#### 3.8.16. Methoxyisogermafurenolide (**16**)

A total of 1.7 mg, white crystals; ${[\alpha ]\;}_{D}^{25}$ -35 (*c* 0.23, MeOH); UV (MeOH) λ_max_ (log ε): 216.8 (4.10); CD: Figure S2; ^1^H-NMR data (CDCl_3_, 600 MHz) in Table S2, ^13^C-NMR data (CDCl_3_, 150 MHz) in Table S3; HRESIMS *m/z* 263.1643 [M + H]^+^ (calcd. for C_16_H_22_O_3_, 263.1642); test purity according to Section 3.7. 97.9%

### 3.9. Cell Culture

#### 3.9.1. Cultivation

Human microvascular endothelial cells (HMEC-1) were provided by Dr. E. Ades und F.-J. Candel (CDC, Atlanta, GA, USA), as well as Dr. T. Lawley (Emory University, Atlanta, GA, USA) and checked free of mycoplasma contamination by PCR and culture from GATC Biotech AG (Konstanz, Germany). The cell line was cultured in EASY Endothelial Cell Growth Medium supplemented with 10% FBS, 50 ng/mL amphotericin B and 50 ng/mL gentamicin in an atmosphere of 5% CO_2_ and 90% relative humidity at 37 °C. Splitting was performed every 2–4 days using trypsin-EDTA. Cells were used for experiments between passage number 3 and 12.

#### 3.9.2. MTT Assay

HMEC-1 cells were seeded in 96-well plates with a density of 9 × 10^4^ cells/well (100 μL/well) and incubated at 37 °C in an atmosphere of 5% CO_2_ and 90% relative humidity for 24 h. Subsequently the medium was removed, sample solution in medium (25–100 µM or 12.5–50 µg/mL) containing a maximum of 0.15% DMSO (*v*/*v*) were added and again incubated for 24 h at the same conditions. After the supernatant was replaced by an MTT solution in medium (0.4 mg/mL) and incubated for 3 h, the cells were treated with 10% SDS in water and stored at room temperature in the dark until the formazan crystals dissolved. The absorbance was determined by a Tecan microplate reader (Tecan Trading AG, Maennedorf, Switzerland) at 560 nm and the viability was calculated as % compared to the average absorbance of the negative control group (only medium). To preclude the possibility of solvent effects, some cells were also treated with the highest used DMSO concentration. All tests was performed *n* = 3 in hexaplicates.

#### 3.9.3. ICAM-1 Assay

This assay was performed as previously described [41]. Therefor confluent grown HMEC-1 cells from a culture flask (150 cm^2^) were split (1:3), suspended in 13 mL medium and seeded in a 24 well plate (500 μL/well). They were cultivated for 48 h at 37 °C in an atmosphere of 5% CO_2_ and 90% relative humidity until they formed a monolayer. Subsequently, the supernatant was replaced by substance solutions in medium (6–100 µM or 12.5–50 µg/mL) containing a maximum of 0.15% DMSO (*v*/*v*) and incubated for 30 min before stimulation with TNFα (10 ng/mL). Each test contained an unstimulated solvent control (0.15% DMSO, *v*/*v*), a negative control (medium) and a positive control (parthenolide, 5 μM); 24 h later, the cells were washed with PBS, detached by trypsin/EDTA, fixed by formalin 10% for 15 min and treated with a murine fluorescein-isothiocyanate (FITC) marked IgG1 antibody against ICAM-1 (Bio-Rad, Kidlington, UK) for 30 min. In case of the extract and HEP fraction the cell suspension (PBS) was analyzed by a Facscalibur^TM^ (BD, Lakes, NJ, USA) (Flow 60 μL/min, FSC: 0.1 V, SSC: 320 V; FITC: 500 V), whereas the analysis of the pure compounds were carried out on a FACSCanto II (BD, Lakes, NJ, USA) (Flow 60 μL/min, FSC: 1 V, SSC: 320 V; FITC: 320 V). The ICAM-1 expression was calculated as % of the mean obtained for the negative control. The test was performed *n* = 3 in duplicates.

### 3.10. Statistics

Significance levels of ICAM-1 expressions were calculated in a one-way Anova followed by Tukey–HSD test using SPSS 26 (IBM, Armonk, NY, USA). IC_50_ levels were obtained using non-linear regression by GraphPad Prism 8.0.0 (GraphPad Software, San Diego, CA, USA).

## Figures and Tables

**Table 1:** ^1^H and ^13^C-NMR data (600 and 150 MHz, respectively; CDCl_3_, *δ* in ppm, *J* in Hz) for compounds **1–3** (s singlet, d doublet, br broad, m multiplet).

No.	1		2		3	
	*δ_H_*	*δ_C_*	*δ_H_*	*δ_C_*	*δ_H_*	*δ_C_*
1	7.02 (1H, d, 7.4)	128.1	7.04 (1H, d, 7.7)	128.3	2.60 (1H, ddd, 6.2, 6.2, 6.2)	52.3
2	7.08 (1H, dd, 7.4, 7.4)	126.7	7.11 (1H, dd, 7.7, 7.7)	127.3	1.69 (1H, dddd, 6.6, 6.6, 6.6, 13.0) 2.05 (1H, dddd, 6.5, 6.5, 6.5, 13.0)	30.4
3	7.02 (1H, d, 7.4)	128.1	7.04 (1H, d, 7.7)	128.3	1.79 (1H, ddd, 6.9, 6.9, 13.5) 1.92 (1H, ddd, 6.9, 6.9, 13.5)	37.0
4		134.1		137.1		95.2
5		137.0		132.5	3.19 (1H, dd, 5.5, 5.5)	66.4
6	3.66 (2H, s)	25.7	3.69 (1H, d, 18.7) 3.80 (1H, d, 18.7)	27.4	4.79 ^1^ (1H, m)	83.4
7		156.2		157.8		161.1
8	4.55 (2H, s)	72.8		105.3	4.64 (2H, s)	72.7
9			1.58 (3H, s)	24.3	4.76 (1H, brs) 4.78 ^1^ (1H, s)	110.1
10		134.0		137.1		146.0
11		123.9		124.7		123.0
12		175.0		171.7		172.8
13	1.43 (3H, s)	11.1	1.29 (3H, brs)	7.5	2.22 (3H, s)	12.9
14	2.27 (3H, s)	20.3	2.28 (3H, s)	20.1	1.76 (3H, s)	21.5
15	2.27 (3H, s)	20.3	2.28 (3H, s)	20.1	1.46 (3H, s)	24.2

^1^ overlapped signal.

**Table 2:** ^1^H and ^13^C-NMR data (400 and 100 MHz, respectively; CDCl_3_, *δ* in ppm, *J* in Hz) for compounds **4** and **5** (s singlet, d doublet, br broad, m multiplet).

No.	4		5	
	*δ_H_*	*δ_C_*	*δ_H_*	*δ_C_*
1	2.23 (1H, ddd, 8.1, 17.5)	47.8		135.4
2	1.68 ^1^ (1H, m) 1.86 ^1^ (1H, m)	25.6	2.19 ^1^ (1H, m) 2.30 ^1^ (1H, m)	28.4
3	1.72 ^1^ (2H, m)	40.8	1.68 ^1^ (2H, m)	39.2
4		81.2		81.0
5	1.73 ^1^ (1H, m)	52.7	2.65 (1H, brd, 11,2)	49.3
6	1.51 (1H, ddd, 7.7, 11.4, 14.1) 1.64(1H, ddd, 5.3, 9.1, 14.2)	27.2	1.51 (1H, ddd, 3.6, 7.8, 14.4) 1.72 ^1^ (1H, m)	27.9
7	2.42 (1H, dddd, 5.3, 7.9, 7.9, 10.9)	44.2	2.26 ^1^ (1H, m)	43.4
8	1.29 (1H, dddd, 2.7, 11.1, 11.1, 13.9) 1.75 ^1^ (1H, m)	26.9	1.65 ^1^ (2H, m)	24.9
9	2.05 (1H, m) 2.53 (1H, ddd, 2.6, 6.6, 14.1)	38.2	2.11 ^1^ (1H, m) 2.25 ^1^ (1H, m)	34.8
10		153.1		127.7
11		86.2		86.3
12	1.41 (3H, s)	23.2	1.44 (3H, s)	23.4
13	1.41 (3H, s)	23.4	1.44 (3H, s)	23.7
14	4.68 (1H, s) 4.70 (1H, s)	106.3	1.55 (3H, s)	20.9
15	1.20 (3H, s)	23.8	1.11 (3H, s)	22.2
1′-OAc		170.5		170.5
2′-OAc	1.97 (3H, s)	22.6	1.97 (3H, s)	22.7

^1^ overlapped signal.

**Table 3:** ^1^H and ^13^C-NMR data for **6** (600 and 150 MHz, respectively; CDCl_3_) and **7** (400 and 100 MHz, respectively; MeOD; *δ* in ppm, *J* in Hz, s singlet, d doublet, q quartet, br broad, m multiplet).

No.	6		7	
	*δ_H_*	*δ_C_*	*δ_H_*	*δ_C_*
1		131.7	5.55 ^1^ (1H, m)	136.5
2	4.30 (1H, dd, 2.9, 2.9)	73.1	5.55 ^1^ (1H, m)	123.6
3	1.60 ^1^ (1H, m) 2.21 (1H, ddd, 2.9, 2.9, 14.4)	31.6	2.79 (1H, dd, 19.2) 2.92 (1H, dd, 19.0)	34.7
4	2.30 (1H, m)	30.3		145.4
5	5.87 (1H, d, 9.4)	74.9	2.18 (1H, dd, 3.3, 13.1)	48.1
6		133.0	2.52 (1H, dd, 13.1, 14.0) 2.85 (1H, dd, 3.5, 14.0)	24.6
7		124.0		164.1
8		153.4	4.97 ^1^ (1H, m)	78.8
9	6.96 (1H, s)	112.9	1.20 (1H, dd, 11.9) 2.37 (1H, dd, 6.6, 11.9)	44.6
10		140.3		37.8
11	3.61 (1H, q, 7.4)	38.9		119.6
12		178.3		175.8
13	1.47 (3H, d, 7.4)	15.5	1.80 (3H, s)	6.7
14	2.39 (3H, s)	19.5	0.98 (3H, s)	18.2
15	1.09 (3H, d, 6.6)	19.1	4.81 (1H, s) 4.99 (1H, s)	106.5
1′-OAc		171.1		
2′-OAc	2.17 (3H, s)	20.9		
1″-Me	3.45 (3H, s)	56.4		

^1^ overlapped signal.

## Data Availability

The data presented in this study are available on request from J.H. and K.K.

## Supplementary Materials

The following are available online: Table S1. ^1^H NMR data of compound **8** (400 MHz; CDCl_3_) and **10–12** (600 MHz; CDCl_3_) (*δ* in ppm, *J in* Hz; s singlet, d doublet, br broad, m multiplet). Table S2. ^1^H-NMR data of compound **13, 15** and **16** (600 MHz; CDCl_3_) (*δ* in ppm, *J* in Hz; s singlet, d doublet, t triplet, br broad). Table S3. ^13^C-NMR data of compound **8** (100 MHz; CDCl_3_) and **10–16** (150 MHz; CDCl_3_) (*δ* in ppm). Figure S1. Influence of the ethanolic extract, HEP fraction, compound **7** (left) and FUR (right) on viability of HMEC-1 cells (MTT assay). The test was performed with pure medium (u.c.), the highest DMSO concentration used in test solutions (0.15%, *v*/*v*) and substance concentrations between 25–100 μM. Data are presented as mean ± SD (*n* = 3). All viability values are situated between 95–100%. Figure S2. CD-spectra of compounds **2–16** in a range of 190–300 nm.

## Sample Availability

Samples of the compounds **1–16** are available from J.H. and K.K.
