# Supplementary material for: Sesquiterpenes from Myrrh and Their ICAM-1 Inhibitory Activity In Vitro
Source: Molecules. 2020 Dec 23;26(1):42. doi: 10.3390/molecules26010042 (PMC7796156; doi:10.3390/molecules26010042)
Supplement: Supplementary file 1 [file molecules-26-00042-s001.pdf]

molecules

Sesquiterpenes from Myrrh and their ICAM-1 Inhibitory Activity *in vitro*

Katrin Kuck [1], Guido Jürgenliemk [1], Bartosz Lipowicz [2] and Jörg Heilmann [1,*]

[1] Institute of Pharmaceutical Biology, Universitätsstr. 31, Regensburg D-93053, Germany
[2] Repha GmbH Biologische Arzneimittel, Alt-Godshorn 87, Langenhagen D-30855, Germany
* Correspondence: joerg.heilmann@chemie.uni-regensburg.de

Supplementary Material

Table S1. ^{1}H-NMR data of compound **8** (400 MHz; $CDCl_3$) and **10–12** (600 MHz; $CDCl_3$) (δ in ppm, J in Hz; s singlet, d doublet, br broad, m multiplet).

no.	8	10	11	12
1	5.53 (1H, d, 8.7)	1.25 (1H, m)	6.87 (1H, d, 9.9)	
		1.58 (1H, m)		
2	5.76 (1H, dd, 5.7, 8.7)	1.64 (2H, m)	6.37 (1H, d, 9.9)	2.45 (1H, ddd, 3.6, 4.1, 15.1)
				2.73 (1H, ddd, 6.0, 15.1, 15.1)
3	5.74[1] (1H, m)	1.97 (1H, ddd, 6.7, 12.5, 12.5)		1.93 (1H, ddd, 5.2, 14.5, 14.9)
		2.37 (1H, ddd, 12.5)		2.03 (1H, ddd, 2.8, 6.0, 13.3)
5	2.32 (1H, m)	1.84 (1H,dd, 3.3, 13.2)		2.89 (1H, s)
6	2.45 (1H, dd, 13.0, 13.0)	2.43 (1H, dd, 13.2, 13.2)		
	2.87 (1H, dd, 3.4, 13.0)	2.63 (1H, dd, 3.3, 13.2)		
9	1.82 (1H, d, 13.8)	1.56 (1H, d, 13.8)	2.94 (1H, d, 16.9)	3.03 (1H, d, 17.9)
	2.28 (1H, d, 13.8)	2.25 (1H, d, 13.8)	3.00 (1H, d, 16.9)	2.95 (1H, d, 17.9)
12			7.17 (1H, s)	7.12 (1H, s)
13	1.84 (3H, brs)	1.83 (3H, brs)	2.27 (3H, brs)	2.20 (3H, s)
14	1.13 (3H, s)	1.03 (3H, s)	1.39 (3H, s)	1.27 (3H, s)
15	1.89 (1H, s)	4.60 (1H, brs)	2.19 (3H, s)	1.73 (3H, s)
		4.87 (1H, brs)		

[1] overlapped signal

Table S2. . ^{1}H NMR data of compound **13, 15** and **16** (600 MHz; $CDCl_3$) (δ in ppm, J in Hz; s singlet, d doublet, t triplet, br broad).

no.	13	15	16
1	5.73 (1H, dd, 10.8, 17.6)	4.68 (1H, d, 12.9)	4.90 (1H, d, 12.9)
2	4.97 (1H, brd, 17.6)	6.17 (1H, d, 12.9)	6.45 (1H, d, 12.9)
	5.00 (1H, brd, 11.0)		
3	4.73 (1H, brs)	4.74 (1H, brs)	4.68 (1H, brs)
	4.99 (1H, brs)	5.01 (1H, brs)	4.80 (1H, brs)
5	2.05 (1H, dd, 4.1, 14.0)	2.00 (1H, dd, 4.1, 13.5)	2.47 (1H,brd, 6.6)
6	2.55 (1H, brt, 14.0, 14.0)	2.53 (1H, dd, 14.0, 14.0)	2.69 (1H, brd, 14.9)
	2.68 (1H, dd, 4.1, 14.0)	2.67 (1H, dd, 3.9, 14.0)	2.72 (1H, dd, 7.1, 14.9)
8	4.83 (1H, dd, 6.2, 12.0)	4.82 (1H, dd, 6.2, 12.0)	4.93 (1H, dd, 6.6, 11.6)
9	1.34 (1H, t, 12.0)	1.31 (1H, dd, 12.1, 12.1)	1.68 (1H, dd, 12.1, 12.1)
	2.21 (1H, dd, 6.2, 12.0)	2.26 (1H, dd, 6.2, 12.1)	2.20 (1H, dd, 6.6, 12.5)
13	1.83 (3H, t, 1.4, 1.4)	1.82 (3H, brs)	1.81 (3H, brs)
14	1.17 (3H, s)	1.15 (3H, s)	1.05 (3H, s)
15	1.77 (1H, s)	1.79 (3H, s)	1.70 (3H, s)
1’-Me		3.50 (3H, s)	3.55 (3H, s)

Table S3. ^{13}C-NMR data of compound **8** (100 MHz; $CDCl_3$) and **10-16** (150 MHz; $CDCl_3$) (δ in ppm).

no.	8	10	11	12	13	15	16
1	137.0	41.3	153.0	210.9	146.5	112.8	111.5
2	122.5	22.3	126.9	34.7	111.8	145.9	147.4
3	120.7	36.1	186.4	38.8	113.9	113.9	113.8
4	136.2	148.5	137.6	70.2	144.9	145.1	147.3
5	48.3	51.7	150.6	62.1	52.9	54.0	51.1
6	23.1	24.5	186.8	195.4	28.4	28.3	28.6
7	160.4	160.7	121.5	119.3	161.9	162.0	161.8
8	103.5	103.3	163.6	165.8	77.9	77.8	78.3
9	48.0	51.3	36.6	35.6	45.7	47.0	40.4
10	35.8	36.7	42.4	51.1	40.9	38.0	37.9
11	122.4	122.3	119.6	119.0	120.1	120.0	120.8
12	172.1	171.9	140.7	140.1	174.9	174.8	174.7
13	8.3	8.2	9.0	8.9	8.2	8.2	8.3
14	15.3	16.6	24.9	20.3	17.0	18.1	28.9
15	20.2	106.9	12.2	23.8	24.7	25.0	25.4
1'-Me						56.1	56.4

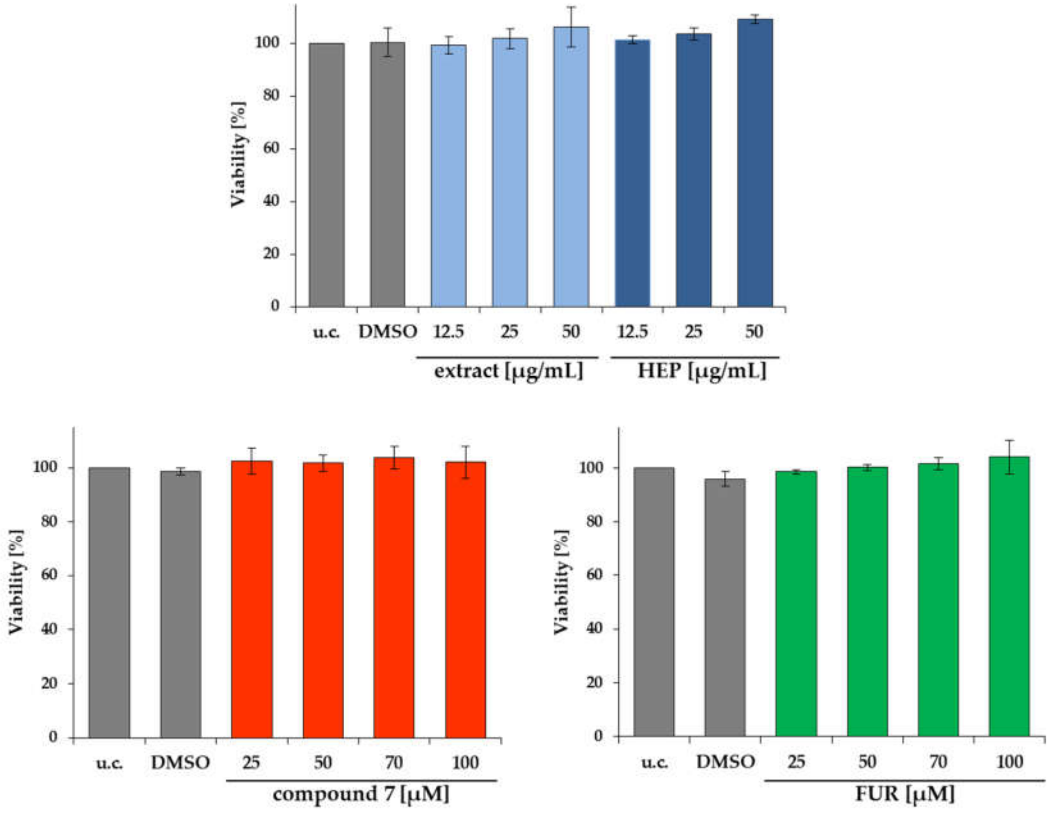

Figure S1. Influence of the ethanolic extract, HEP fraction, compound **7** and FUR on viability of HMEC-1 cells (MTT assay). The test was performed with pure medium (u.c.), the highest DMSO concentration used in test solutions (0.15%, *v*/*v*) and substance concentrations between 25–100 µM. Data are presented as mean +/- SD (n = 3). All viability values are situated between 95–100%.

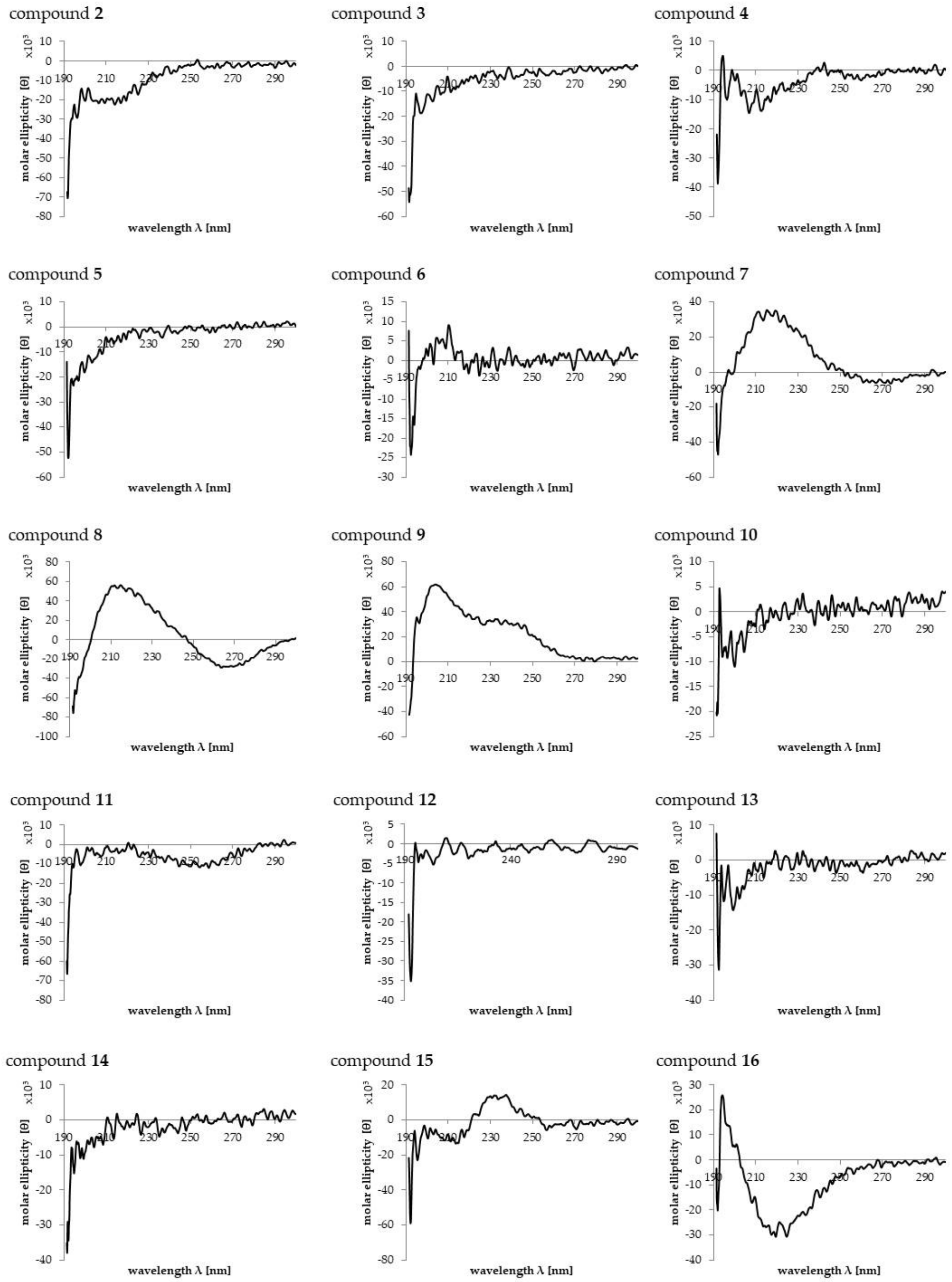

Figure S2. CD-spectra of compounds **2–16** in a range of 190–300 nm.
